# Rationalism vs. Radicalism*Read before the Indianapolis Dental Society, April, 1921, published in “Dental Summary,” June, 1922.

**Published:** 1923-01

**Authors:** F. R. Henshaw


### RATIONALISM VS. RADICALISM*

BY F. R. HENSHAW, D.D.S.

Little did the fathers of dentistry, Hayden, Harris, Parmelee, Brown and their confreres guess of the problems that the profession they were so generously endowing with their skill and learning, was to meet and puzzle over within less than a century after their day. When these men wrought, only those mechanical procedures incident to the restoration of lost tooth substance or dental members demanded their skill, and most wonderfully did they perform these operations with the limited armamentarium at their command.

The succeeeding generations of dentists found their labors becoming more and more complicated, until at the present time we have arrived at a place where all the sciences must be brought to the aid of the man who aspires to give to his clients that finished product of service, which shall co-ordinate with the work of the physician, surgeon, internist and hygienist.

In arriving at our present state of practice we have undergone many spasms of radical thought, some of which were productive of much good as well as of much harm both to the profession and to the public. Always it has been the tendency of mankind, especially we of America, to be faddists. No one has ever propounded a new creed or cult with vigor and zeal, and such persons are always vigorous and zealous, without attracting followers who go even further than the originators intended or expected to go. Whether it be in religion, politics, temperence or science the result has always been the same; some radicals are always to be found who are not deterred by those natural inhibitions which are so essential to the maintenance of the balance of sanity and good judgment.

---

*Read before the Indianapolis Dental Society, April, 1921, published in "Dental Summary," June, 1922.

We wouldn't know what to do without them but sometimes we don't know what to do with them.

We are all faddists to a greater or lesser degree. This has been demonstrated many times in the profession of dentistry.

One of the early fads, beginning in the early 90's was the crown and bridge-work fad which spread like wildfire to every city, village and farm, until the gold-shell crown shone like a headlight at every turn. Here again the radical overplayed his hand, and because of the careless, inefficient and oftentimes dishonest methods of construction employed, finally brought this type of work into such disrepute that it called forth the much resented but highly-merited rebuke of Hunter, the English investigator, who brought the whole dental profession up standing by his startling and authentic statements. From that time a spirit of conservatism has consistently prevailed in the construction and application of crowns and bridges, that has done much to remove the just reason for criticism that before existed.

To be sure there are those who have gone to the other extreme in the past year or two, and utterly condemned and abandoned the fixed bridge in any form in favor of the so-called removable bridge, but there is already indication that there is a return of sentiment in favor of the rational use of both, as best indicated in the particular case, and that neither is a panacea for all ills. Certainly the day of the fourteen-tooth bridge is past, but just as certainly many penalties are to be paid for the removable bridge.

For many years prior to about 1903, dentists had constructed occasional porcelain inlays in well-selected cases, and had thereby made beautiful cosmetic restorations that commanded the admiration of all who saw them.

Suddenly, like a clarion call, came the porcelain fad. Every dentist in the profession suddenly became a porcelain expert over night and the literature of that day contains little else. The fad continued at fever heat until it began to dawn upon the profession, and the public as well, that the results were not as expected. Then came the ebb and like a tide going out it left the porcelain art stranded high and dry. Now it was not because of the lack of practicability of the porcelain inlay that this fad exploded, but purely and simply because of the lack of that fine technical training and experimentation that are absolutely essential to success with this material. This is borne out by the fact that the tenacious, rational porcelain workers who survived the crash, are today doing exactly the things that we all hoped and tried to do in the days of the porcelain rush.

Then the root-canal fad. With the advent of pressure anesthesia it became a very simple matter to painlessly destroy the pulps of teeth, and as that eliminated present pain in our immediate operations, everyone did it gladly and gleefully, secure in the belief that we could so perfectly fill those root canals as to preclude the possibility of future trouble.

Some of us went so far as never to place a crown on a tooth without first devitalizing, and at least one man had the reputation of never filling a tooth until he had first removed the pulp and filled the canal. All this covered a long period of time and tens of thousands of teeth were thus treated.

Then came the X-ray picture and the discovery of focal infection, and every good, conscientious operator was thrown into consternation by the findings reported by the pathologist and bacteriologist. No one could be sure of himself nor of his methods of practice, nor could he trust the advice of his neighbor and friend.

No more serious dilemma was ever encountered by any profession than this, for at once the radical began to demand the removal of every tooth in which the pulp had been destroyed, and the medical profession, seeing in these focal infections a possible solution of many of their puzzling and obscure cases, added to the score by ordering their patients to have whole rows of teeth removed. There is not the slighest doubt that many afflictions of obscure origin have been cleared up by the removal of oral foci of infection, but also there is not the slightest doubt that thousands of teeth have been unnecessarily sacrificed without the slightest benefit to the patient. The best minds of the dental and medical professions are working on the solution of this grave and difficult problem, and until the evidence is all in and carefully weighed and sifted, it behooves us, as a profession, to keep our heads and maintain a conservative position in these matters not only for the sake of our profession, but for the sake of the public who become the ready victims of these wild impulses.

The radiograph has not proven to be an unmixed blessing. In no other field is there greater reason for careful study and wide information and even in the most skillful hands there is chance for grievous error.

To my mind the most accomplished dental radiographer in America is our own Dr. Howard R. Raper who has devoted the principal part of his life to this work.

From the very beginning Dr. Raper has recognized the shortcomings of the dental radiograph, and in all his writings he has counseled a conservative interpretation and thoughtful diagnosis. The principal danger arising from the use of the dental radiograph lies in the half-baked diagnosis that is so frequently rendered upon the radiograph alone, without due and proper consideration of all other co-related physical signs and symptoms.

The most important need for consultation with the physician and internist arises in consideration of the dental radiograph as a diagnostic agent, for without a definite knowledge of the general physical condition of the patient the value of the dental radiograph becomes greatly depreciated.

Perhaps no wider divergence of opinion exists than in the diagnosis of so-called pyorrhea, or to use the term invented by the American Society of Periodontists, periodontoclasia; yet there can hardly be justification in pronouncing cases of simple gingivitis that can be cleared up by the usual well-known means of prophylaxis, as serious cases of pyorrhea. Yet just this thing is being done, to the consternation, fright and mental anguish of many victims who are entitled at least to a fair statement of their mouth conditions.

Within a short time the dental profession will receive a publication showing exact measurements and figures and adequate models, demonstrating that the practice recently in vogue of removing great masses of the alveolar border by the operation known as alveolectomy in cases of extensive extraction, leaves the mouth in such a deformed condition as to utterly preclude the best and most proper scientific prosthetic restoration. This has been known as a radical operation and has much to commend it in the rapidity with which the tissues heal, but it will not stand the test of being to the best interest of the patient in the long run, and needs the gentle hand of the conservative to check it and relieve it of its serious objections.

In dental education of the public there have been great strides, but fortunately it has been largely along safe and sane channels and by men who have had the breadth of vision to put the facts in such simple form that the public has begun to appreciate the great work that is being done by the dental profession.

No profession has been more generous and unselfish in its treatment of the cause of the children, and at all times the heartiest co-operation has been manifested in all of the public health movements, so that today we stand shoulder to shoulder with our medical brethren in helping to build up a better and stronger race.

In the dental colleges the courses have been so standardized of recent years, that no just cause for complaint against any of the recognized schools can be said to exist at the present time. The advance from a three to a four-year course has brought about a higher development of the student and has made of him a more finished product. In spite of the fact that the four-year course is just now giving us its first finished product, there has been a very great effort on the part of some of the more radical leaders to force all schools into a requirement of five years beginning in 1921, and six years beginning in 1925.

Whatever virtue there may be in this demand is more than offset by the fact that if all the colleges in America had twice their present capacity, they would not be able in the period of the next ten years to produce enough dentists to supply the demand that has been created for dental service by the wide-spread Oral Hygiene campaign carried on for the past decade. The calmer, more conservative heads in the dental teaching profession have wisely counseled that we make haste slowly, and the schools, members of the National Association of Dental Faculties, will not inaugurate the additional year at the present time. It is felt that the needs of the public and of the dental profession will be best served by a slow and steady progress rather than by swift leaps and bounds.

Perhaps it may seem from what I have said that I feel that the profession is going to the demnition bow-wows, but I assure you that my feeling is quite the contrary. I feel that dentistry is on safe ground and in safe hands, and I do not wish to detract from the virtues of the men who are radical, for they are necessary to the progress that is being made, and without them we would probably stagnate, and so long as the great mass of us remain rational we shall profit and prosper.

Finally permit me to submit one further thought:

There is a class of men in our ranks who are ashamed of their profession and of their degree of Doctor of Dental Surgery, and who would, if it were within their power, require that every man entering the practice of dentistry secure his training in a medical school and become a dental specialist with the degree of Doctor of Medicine. The experience of the dental profession in working out its own salvation as an independent profession has been productive of all the good that has grown up with us, and the contrary has proven to be the case in those countries where the Medical School has dominated; so of all the radicals that I

hope to be protected from, that one most of all is he who is ashamed of his profession and who would like to put away the good and honorable degree of Doctor of Dental Surgery.

